# Supplementary material for: CCL11 Differentially Affects Post-Stroke Brain Injury and Neuroregeneration in Mice Depending on Age
Source: Cells. 2019 Dec 26;9(1):66. doi: 10.3390/cells9010066 (PMC7017112; doi:10.3390/cells9010066)
Supplement: Supplementary file 1 [file cells-09-00066-s001.pdf]

Table S1: Experimental groups of adolescent male mice

	CCL11	PBS	SB297006	DMSO
TTC d1	n = 10	n = 10		
TTC d3			n = 9	n = 10
IHC d1	n = 10	n = 10		
IHC d3			n = 9	n = 10
IHC d28	n = 8	n = 10		
FC d7	n = 5	n = 5		
WB d7	n = 5	n = 4		
Behavorial tests d28	n = 8	n = 10		

Abbreviations: TTC, triphenyltetrazolium chloride staining; IHC, immunohistochemistry; FC, flow cytometry; WB, western blot

Table S2: Experimental groups of adult male mice

	CCL11	PBS	SB297006	DMSO
TTC d1	n = 7	n = 8		
TTC d3			n = 12	n = 8
IHC d1	n = 7	n = 8		
IHC d3			n = 12	n = 8
IHC d28	n = 6	n = 7		
FC d7	n = 4	n = 4		
WB d7	n = 4	n = 4		
Behavorial tests d28	n = 6	n = 7		

Abbreviations: TTC, triphenyltetrazolium chloride staining; IHC, immunohistochemistry; FC, flow cytometry; WB, western blot

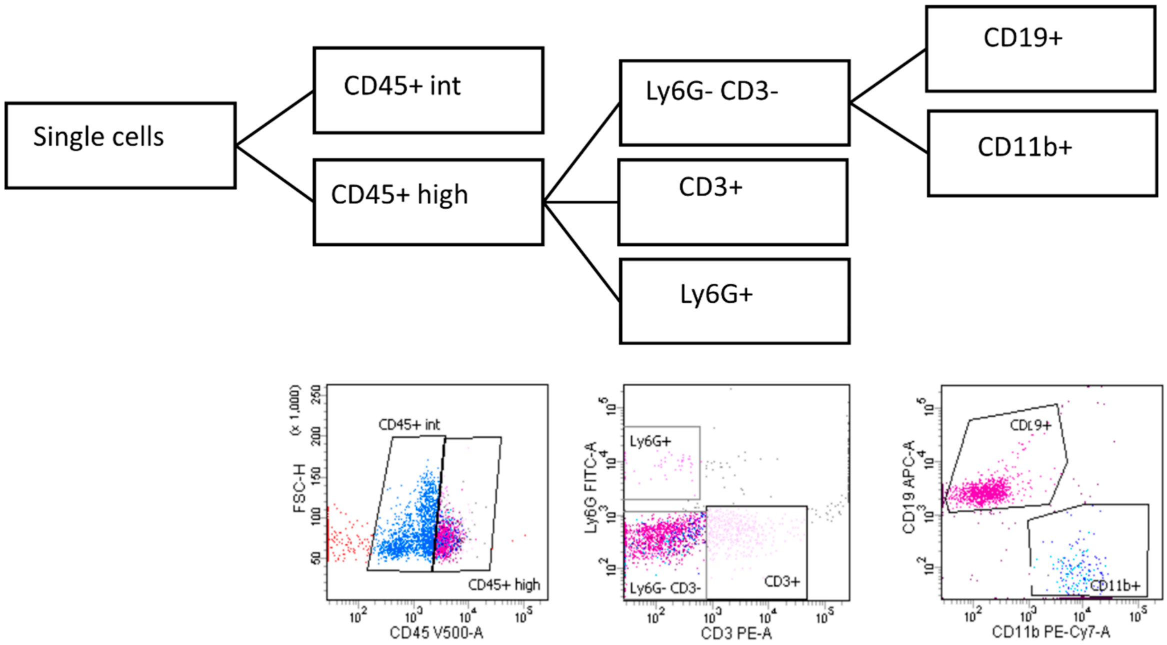

Figure S1: Flow cytometry gating strategy. C57BL6 male mice were subjected to experimental stroke for 45 minutes. With reperfusion they received 10 µg/ kg Bodyweight CCL11 or 100 µl PBS as control intraperitoneal and daily for 7 days. Flow cytometry was analyzed with FlowJo software at day 7 after stroke. The subsets of leukocytes ($CD45^{high}$), microglia ($CD45^{intermediate}$), T cells ($CD45^{high}CD3^{+}$), neutrophils ($CD45^{high}Ly6G^{+}$), B cells ($CD45^{high}CD3^{-}Ly6G^{-}CD19^{+}$) and dendritic cells ($CD45^{high}CD3^{-}Ly6G^{-}CD11b^{+}$) were analyzed. Below scatter plot graph at each stage of gating strategy.

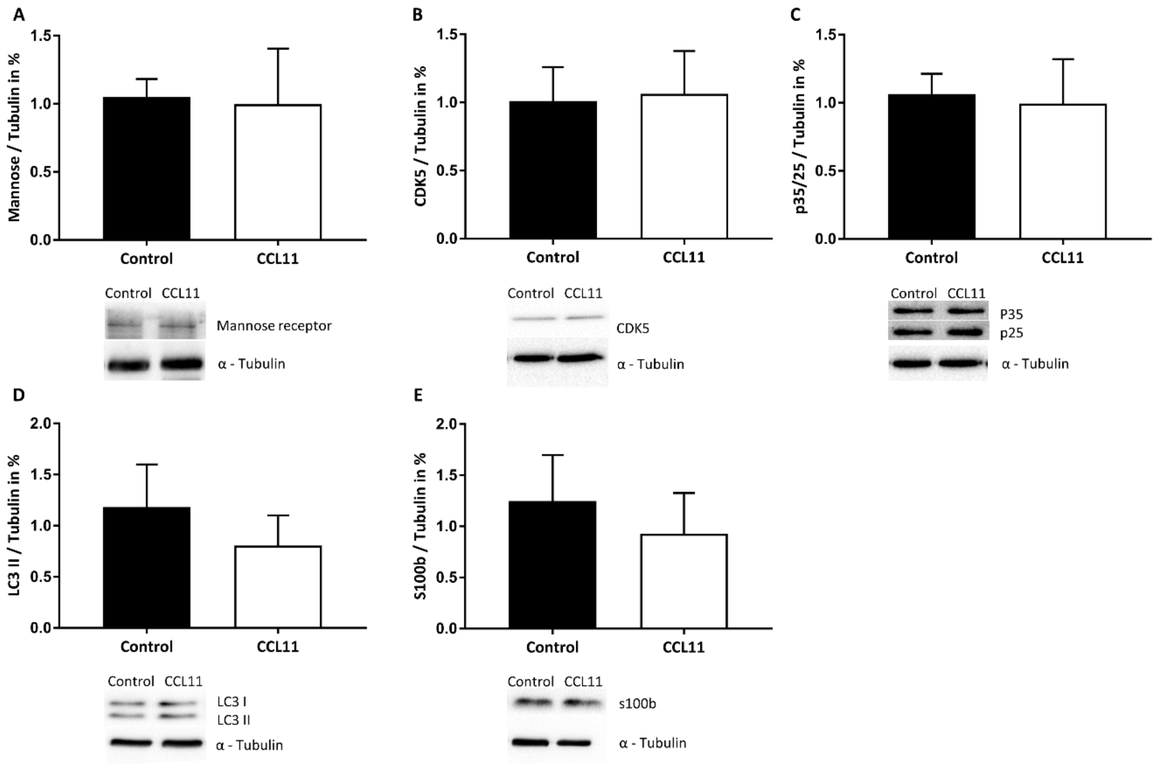

Figure S2: CCL11 does not affect autophagy-related signaling pathways in adolescent mice. C57BL6 male mice with an age of six weeks were subjected to cerebral ischemia for 45 min followed by reperfusion for 7 d. At the beginning of the reperfusion, mice received intraperitoneal injections of either 10 µg/kg bodyweight of CCL11 or 100 µl of PBS as control followed by daily additional injections on the seven consecutive days after reperfusion. Demonstrated is the quantitative analysis of (A) mannose receptor, ns p = 0.896, (B) CDK5, ns p = 0.648, (C) p25/p35, ns p = 0.321, (D) LC3 II, ns p = 0.083 and (E) s100b, ns p = 0.112, proteins by Western blotting (using alpha tubulin as loading control, representative blots are shown below each diagram) of 6-week old mice. n = 5 in the CCL11 group and n = 4 in the control group. All data are given as means ± S.D.

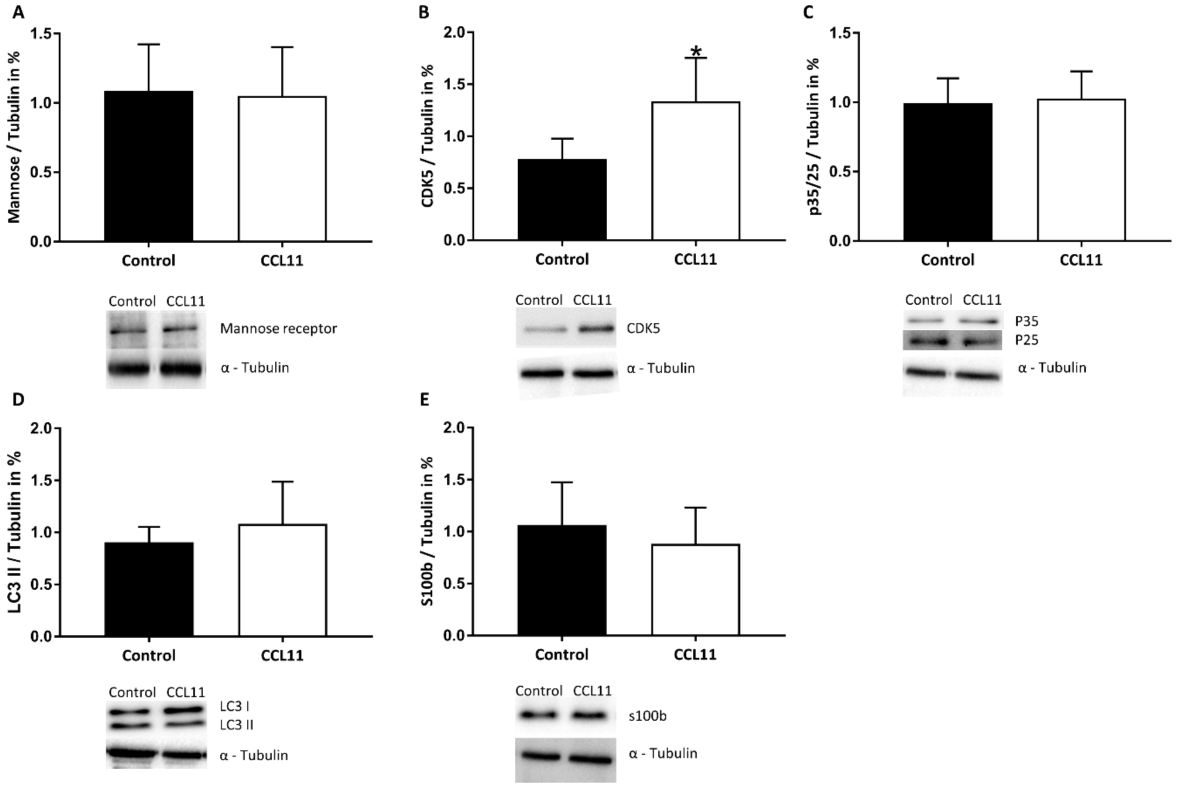

Figure S3: CCL11 does not affect autophagy-related signaling pathways in adult mice. C57BL6 male mice with an age of six months were subjected to cerebral ischemia for 45 min followed by reperfusion for 7 d. At the beginning of the reperfusion, mice received intraperitoneal injections of either 10 µg/kg bodyweight of CCL11 or 100 µl of PBS as control followed by daily additional injections on the seven consecutive days after reperfusion. Demonstrated is the quantitative analysis of (A) mannose receptor, ns p = 0.878, (B) CDK5, *significantly different from the corresponding control, p = 0.028, (C) p25/p35, ns p = 0.878, (D) LC3 II, ns p = 0.798, and (E) s100b, ns p = 0.278, proteins by Western blotting (using alpha tubulin as loading control, representative blots are shown below each diagram) of 6-month old mice. n = 4 in each the groups. All data are given as means ± S.D.
